# Inhibition of ecto-5′-nucleotidase and adenosine deaminase is able to reverse long-term behavioural effects of early ethanol exposure in zebrafish (*Danio rerio*)

**DOI:** 10.1038/s41598-020-74832-0

**Published:** 2020-10-20

**Authors:** Aline Haab Lutte, Julia Huppes Majolo, Rosane Souza Da Silva

**Affiliations:** Laboratório de Neuroquímica E Psicofarmacologia, Escola de Ciências da Saúde E da Vida, Pontifícia Universidade Católica Do Rio Grande Do Sul, Avenida Ipiranga, 6681, Porto Alegre, RS 90619-900 Brazil

**Keywords:** Disease model, Embryogenesis, Reprogramming, Biochemistry, Neuroscience

## Abstract

The behavioural impacts of prenatal exposure to ethanol include a lower IQ, learning problems, anxiety and conduct disorders. Several components of the neurochemical network could contribute to the long-lasting effects of ethanol embryonic exposure. Adenosine is an important neuromodulator, that has been indicated to be affected by acute and chronic exposure to ethanol. Here, embryos of zebrafish exposed to 1% ethanol during the developmental stages of gastrula/segmentation or pharyngula exhibited anxiolytic effect, increased aggressiveness, and decreased social interaction. The exposure during pharyngula stage was able to affect all behavioural parameters analysed at 3 months-post fertilization (mpf), while the treatment during gastrula stage affected the anxiety and social interaction parameters. The aggressiveness was the only behavioural effect of early ethanol exposure that lasted to 12 mpf. The use of a specific inhibitor of adenosine production, the inhibitor of ecto-5′-nucleotidase (AMPCP/150 mg/kg), and the specific inhibitor of adenosine degradation, the inhibitor of adenosine deaminase, EHNA (100 mg/kg) did not affect the effects over anxiety. However, AMPCP at 3 mpf, but not EHNA, reversed aggressive parameters. AMPCP also recovered the social interaction parameter at 3 mpf in animals treated in both stages, while EHNA recovered this parameter just in those animals treated with ethanol during the gastrula stage. These results suggest that long-lasting behavioural effects of ethanol can be modulated by intervention on ecto-5′-nucleotidase and adenosine deaminase activities.

## Introduction

Worldwide, the percent of pregnant women who consume ethanol via alcoholic beverages is estimated to be around 10%^1^. The term alcohol-related neurodevelopmental disorder (ARND) describes a particular pattern of disordered behaviour and impaired cognitive development in children and young people who did not develop physical and morphological features related to alcohol exposure^2^. The behavioural impacts of prenatal exposure to ethanol include a lower IQ, learning problems, anxiety and conduct disorders^3,4^. Several animal models, especially rodent-based models and more recently zebrafish models, were developed to contribute to a trustworthy picture of the neurobiology of the behaviour sequelae of ARND^5–7^. The cellular targets underlining the long-lasting cognitive and behavioural outcomes of ethanol exposure include components of serotoninergic, GABAergic, glutamatergic and dopaminergic systems. Data from human and animal studies indicate that ethanol exposure during early brain development is able to block NMDA receptors, to exacerbate GABA_A_ receptor action, to decrease 5-HT-binding and to decrease dopamine levels^8–11^.

Besides the role of adenosine in energy metabolism, epigenetic mechanisms and the genetic transmission of information, this nucleoside exerts an important neuromodulatory effect^12,13^. Adenosine is able to affect the synaptic activity by pre-, post- and non-synaptic actions, especially through control of neurotransmitter release, receptor-receptor interaction and post-synaptic impact on membrane depolarization^14,15^. The ability of adenosine to modulate the major neurotransmitter systems related to common cognitive and behavioural consequences of early ethanol exposure brings the attention to the possible involvement of this nucleoside on these long-term effects. The activation of adenosine receptors and, therefore, the adenosine neuromodulation, are a reflection of the control of the extracellular levels of this nucleoside, which are a result of several enzymatic activities, such as ecto-5′nucleotidase and ecto-adenosine deaminase, and of equilibrative and concentrative transport mechanisms that control the availability of adenosine for the P1 receptors and, consequently, the neuromodulation of major neurotransmitter systems^16^. The ecto-adenosine deaminase (E.C. 3.5.4.4) catalyzes the deamination of adenosine producing inosine and NH_3_^17^. The ecto-5′-nucleotidase (E.C. 3.1.3.5) catalyzes the dephosphorilation of AMP producing adenosine and inorganic phosphate^18^. In fact, the purinergic system is also a direct target of ethanol exposure^19–24^. Both enzymes are affected by acute or chronic exposure to ethanol in mature CNS^22,23^.

Behaviour impairments caused by ethanol have been widely studied over the years in the zebrafish (*Danio rerio*) model^25–27^. The embryonic zebrafish has some advantages as a model of early ethanol exposure, since zebrafish shows easy manipulation and clear differentiation of the developmental stages through a transparent egg^28^. Zebrafish exposed to alcohol concentrations ranging between 0.25 and 1.00% shows no apparent morphological changes^29^. The use of doses above 1% may cause significant decrease in survival and morphological deformities such as pericardial edema, axial malformations, yolk sac edema, cyclopia^30^ and micropthalmia^30–32^. In that sense, in order to give base to studies of subtle effects of ethanol that could contribute to behavioural effects, the use of doses bellow 1% ethanol is suitable. Zebrafish neuroanatomy has many similarities to that of mammals, especially in areas such as the spinal cord and hindbrain, in which zebrafish and mammals share major neurotransmitter and neuromodulator pathways, including the expression and functionality of major enzymes and receptors of purinergic system^33–35^. An increasing number of studies have demonstrated that ethanol exposure affects a variety of zebrafish behaviours, such as anxiety^36^, aggressiveness^25,37^, memory^38^ and social interaction^39^, some of which are long-lasting^38,39^. Therefore, because adenosine is a potent neuromodulator and ethanol is able to affect several neurotransmitters modulated by adenosine and the purinergic system itself, the objective of this research was to investigate and understand the possible effects of interventions on enzymes involved in adenosine production and degradation over the long-lasting behavioural effects caused by ethanol exposure in different stages of early development.

## Results

**Figure 1:**
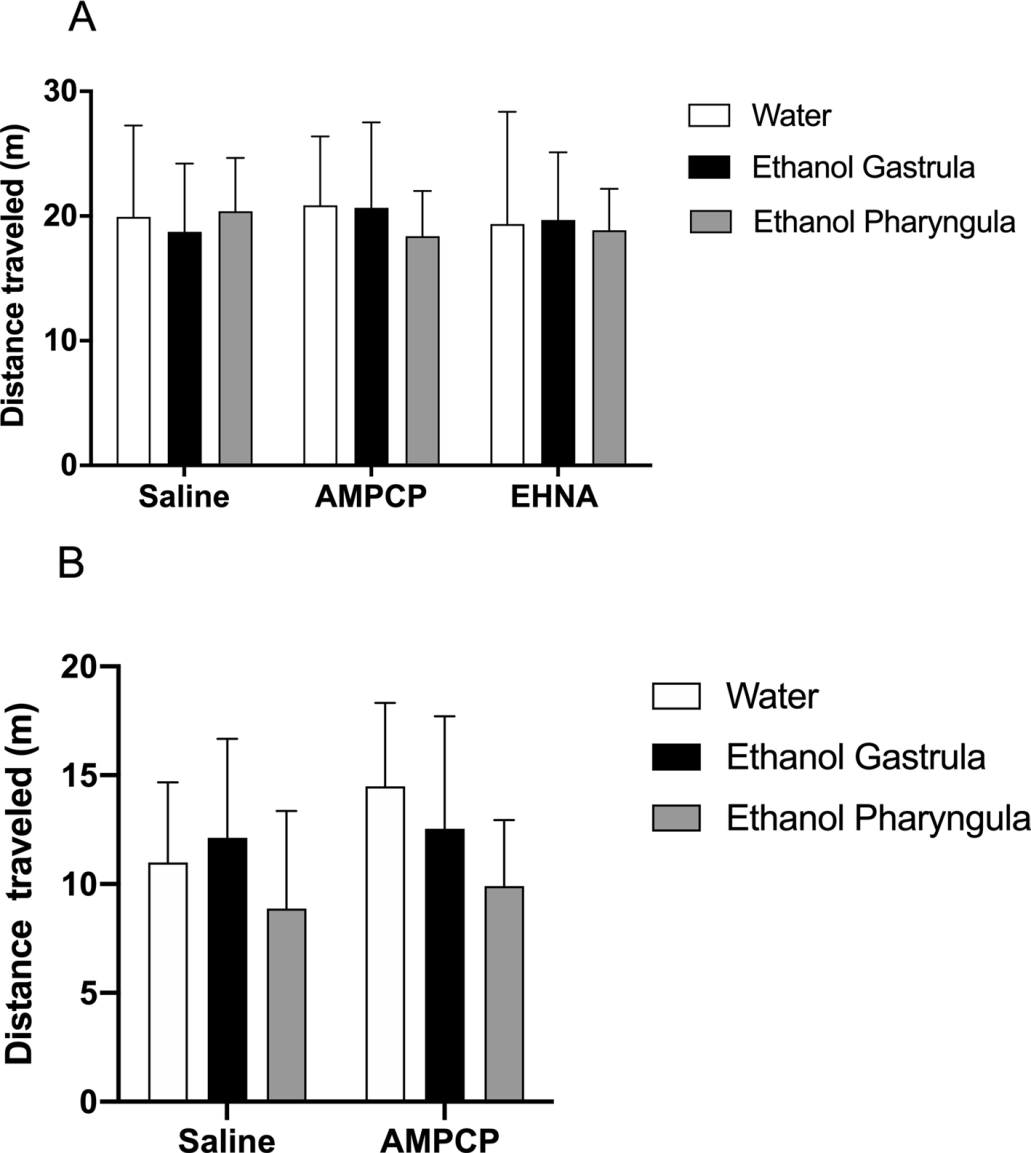
The locomotor parameter distance travelled (m) evaluated at 3 mpf (**A**) and 12 mpf **(B)** in animals treated with 1% ethanol during the gastrula/segmentation or pharyngula stages. AMPCP or EHNA inhibitors alone did not generate significant effects on any parameter or period of ethanol exposure when compared to the control/saline group. Data were expressed as mean ± SD and analyzed by two-way ANOVA. Multiple analysis of group was performed using Tukey’s test (n = 10 animals per group per experiments).

**Figure 2:**
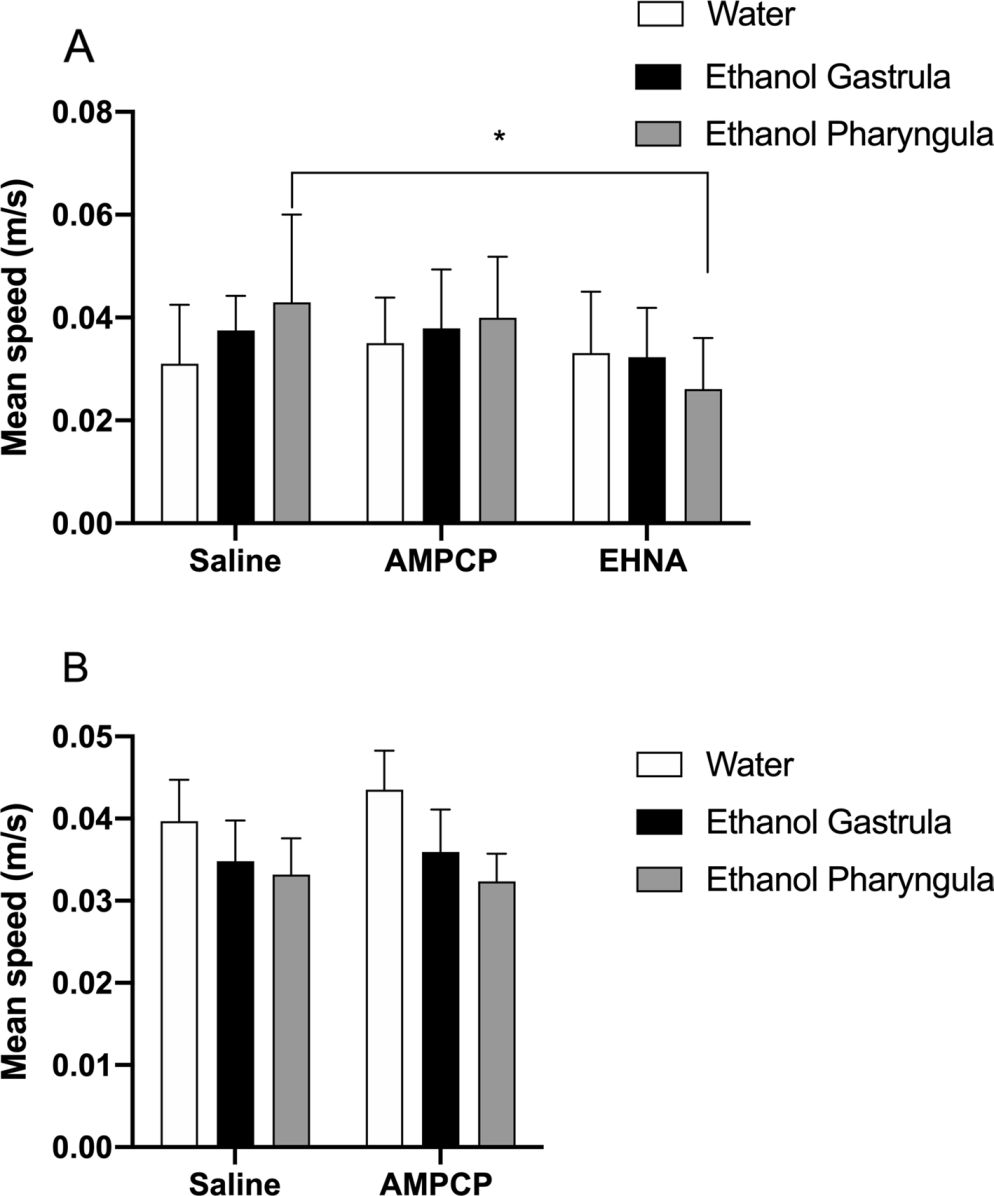
The locomotor parameter mean speed (m/s) evaluated at 3 mpf (**A**) and 12 mpf **(B)** in animals treated with 1% ethanol during the gastrula/segmentation and pharyngula stages. AMPCP and EHNA inhibitors alone did not generate a significant effect on any parameter or period of ethanol exposure when compared to the control/saline group. EHNA inhibitors had a mild effect in mean speed, when used at 3 mpf animals treated with ethanol in the pharyngula stage (**A**), in comparison to the ethanol/saline treated animals (*P < 0.05). **(B)** No effect of ethanol treatment was detected at 12 mpf. Data were expressed as mean ± SD and analyzed by two-way ANOVA. Multiple analysis of group was performed using Tukey’s test (n = 10 animals were randomized assigned for experiments).

Animals treated with 1% ethanol during gastrula/segmentation or pharyngula stages did not show altered morphological (data not shown) or survival rate (data not shown) outcomes when evaluated at 3 or 12 mpf. Locomotor parameters (distance travelled and mean velocity) had few effects of the factors evaluated (Figs. 1, 2). At 3 mpf, the distance travelled was not affected (Fig. 1A), while the mean velocity had significant effect of factor *purinergic intervention* [F_(2;81)_ = 3.75; *P* = 0.028] (Fig. 2A). EHNA administration significantly reduced the mean velocity of animals treated with 1% ethanol at pharyngula at 3 mpf (*P* = 0.03) (Fig. 2A). At 12 mpf, the factor *ethanol treatment* had significant effect on distance travelled [F_(2;54)_ = 3.81; *P* = 0.028] (Fig. 1B), while no effect was identified in mean velocity (Fig. 2B). The inhibitors of ecto-5′-nucleotidase (AMPCP) and adenosine deaminase (EHNA) alone did not generate a significant effect on any locomotor parameter when compared to the water/saline group (Figs. 1, 2).

**Figure 3:**
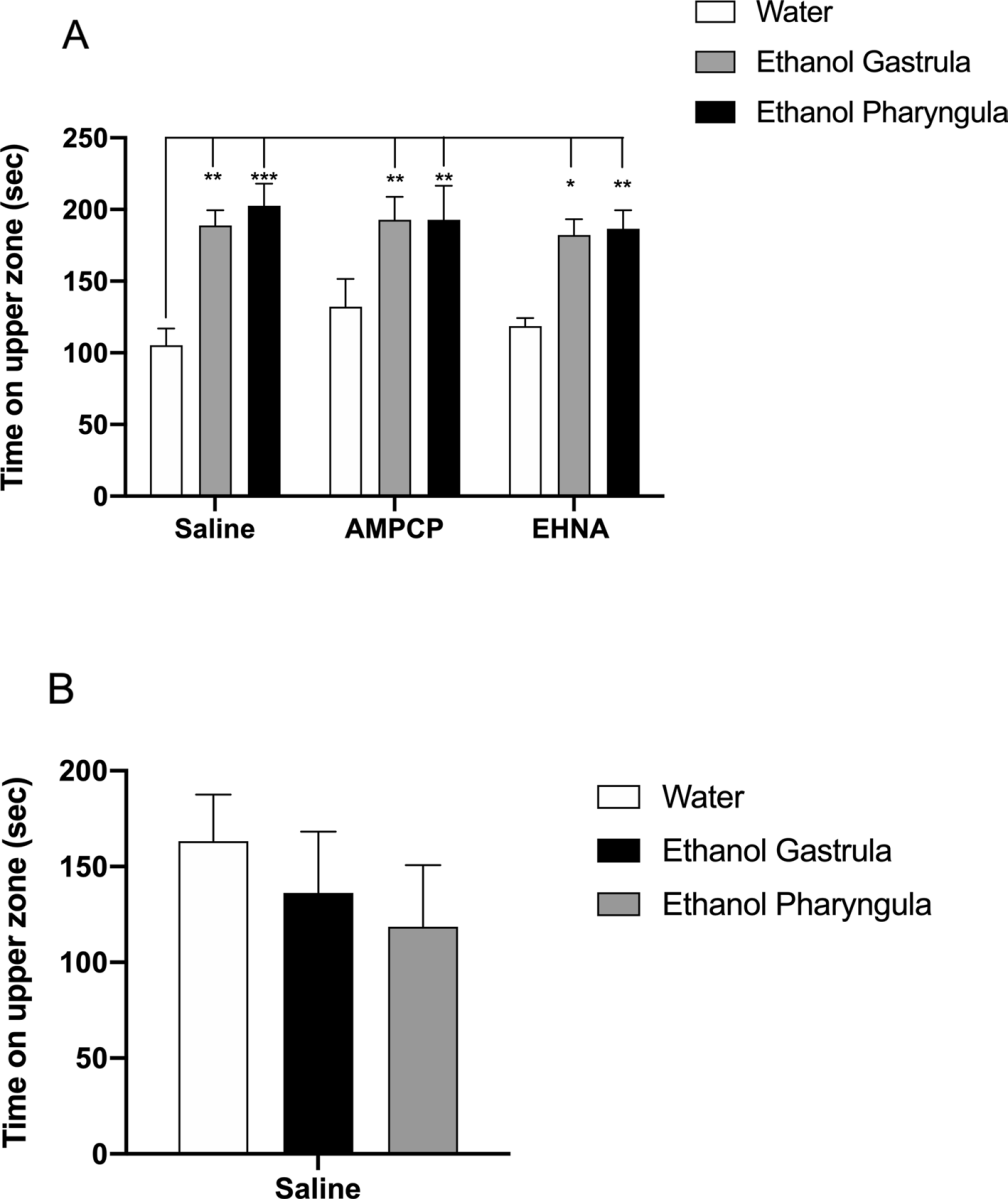
Long-term effects of 1% ethanol treatment during gastrula/segmentation or pharyngula stages on the time spent in the upper zone (seconds) at 3 mpf (**A**) and 12 mpf **(B)**. At 3 mpf, ethanol increased the time spent in the upper zone for all groups (*P < 0.05; **P < 0.01; ***P < 0.001) in comparison to control group (water/saline). AMPCP or EHNA inhibitors alone did not generate a significant effect on any parameter or period of ethanol exposure when compared to the control/saline group. Time spent in the upper zone was registered for 5 min by video recording in the tank diving behavioural test. Data were expressed as mean ± SD and analyzed by two-way ANOVA for 3 mpf and one-way ANOVA for 12 mpf. Multiple analysis of group was performed using Tukey’s test (n = 10 animals were randomized assigned for experiments).

Zebrafish treated with 1% ethanol during gastrula/segmentation or pharyngula stages had long-lasting behavioural effects. To check the behaviour related to anxiety, the time the zebrafish spent in the upper zone of the aquarium was analysed. At 3 mpf, the factor *ethanol treatment* had significant effect over time in the upper zone [F_(2;81)_ = 23.48; *P* < 0.0001]. Ethanol treatment in both developmental stages produced an anxiolytic effect, since the 3 mpf animals increased their time in the upper zone by 79% in the gastrula/segmentation group and 92% in the pharyngula group, compared to water/saline group (*P* = 0.0049 and 0.0005, respectively; Fig. 3A). Neither EHNA nor AMPCP treatment altered the ethanol effects over time in the upper zone of aquarium when compared to water/saline group at 3 mpf (Fig. 3A). At 12 mpf, there was no alteration of time spent in upper zone between experimental groups [F_(2;27)_ = 0.5721; *P* = 0.57] (Fig. 3B).

**Figure 4:**
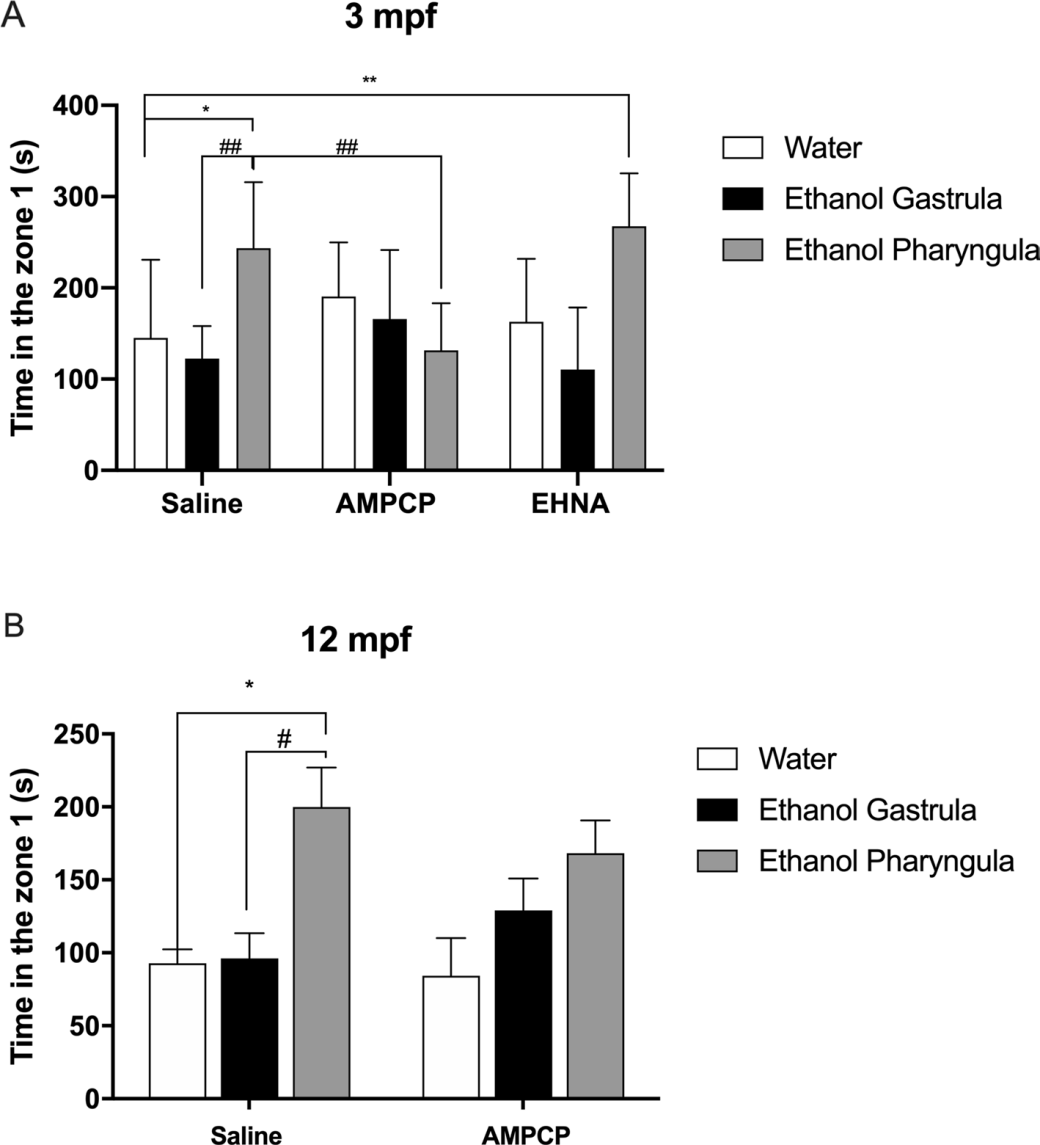
Long-term effects of 1% ethanol treatment during gastrula/segmentation or pharyngula stages on aggressive behaviour at 3 mpf (**A**) and 12 mpf **(B)**. Treatment with ethanol during the gastrula/segmentation stage and the use of AMPCP or EHNA inhibitors alone did not generate a significant effect compared to the control/saline group. (**A**) At 3mpf, the 1% ethanol treatment during pharyngula stage caused an increase in aggressive behavior in comparison to control group (water/saline) (*P < 0.05) and ethanol-treated animals at gastrula/segmentation stage receiving saline (##*P* < 0.01). This effect was recovered by AMPCP at 3 mpf (##*P* < 0.01). **(B)** At 12 mpf, the 1% ethanol treatment during pharyngula stage caused an increase in aggressive behavior in comparison to control group (water/saline) (*P < 0.05) and ethanol-treated animals at gastrula/segmentation stage receiving saline (#*P* < 0.05). Data were expressed as mean ± SD and analyzed by two-way ANOVA. Multiple analysis of group was performed using Tukey’s test (n = 8–10 animals were randomized assigned for experiments). Zone 1 = the nearest zone to the mirror.

There is a significant contribution of the factor *ethanol treatment* to the time spent in the zone 1 (zone nearest to the mirror), a measure to assess aggressiveness, at 3 mpf animals [F_(2;79)_ = 11.94; *P* < 0.0001] (Fig. 4A). However, a significant interaction between factors was identified by the statistical analysis [F_(4;79)_ = 7.597; *P* < 0.0001]. At 3 mpf, the time spent in the zone 1 was significantly increased in animals treated with 1% ethanol during pharyngula stage when compared to control/saline animals (*P* = 0.048) and animals treated with 1% ethanol during gastrula/segmentation stage (*P* = 0.002) (Fig. 4A). This increased behaviour related to aggressiveness registered in 3 mpf animal treated with 1% ethanol during pharyngula stage was reduced by AMPCP (*P* = 0.0065), but not by EHNA (Fig. 4A). At 12 mpf, there is also a significant contribution of the factor *ethanol treatment* to the time spent in the zone 1 [F_(2;61)_ = 10.31; *P* = 0.0001]. The time spent in the zone 1 was significantly increased in 12 mpf animals treated with 1% ethanol during pharyngula stage when compared to control/saline animals (*P* = 0.014) and animals treated with 1% ethanol during gastrula/segmentation stage (*P* = 0.015) (Fig. 4B). This increased time in zone 1 registered in 12 mpf animal treated with 1% ethanol during pharyngula stage was not affected by AMPCP (Fig. 4B). EHNA was not tested at 12 mpf animals since had no effect at 3 mpf animals. Additional observation (Supplementary Table S1) indicated that animals treated with ethanol during pharyngula stage exhibited the behaviours of biting, sprinting and changes in colour pattern, considered indicators of aggressive behaviour^40^.

**Figure 5:**
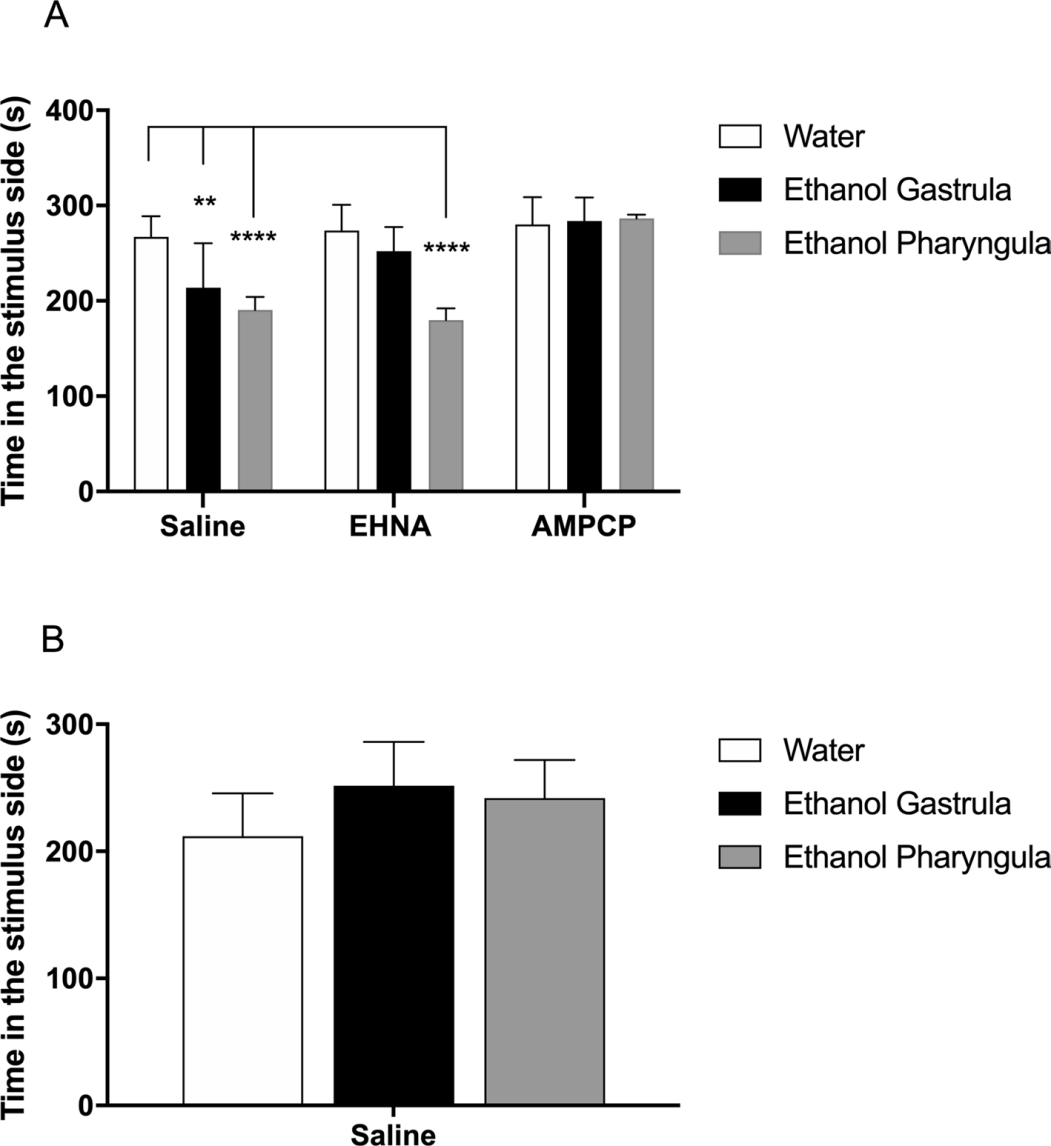
Long-term effects of 1% ethanol treatment during gastrula/segmentation or pharyngula stages on social interaction behaviour at 3 mpf (**A**) and 12 mpf **(B)**. The use of AMPCP or EHNA inhibitors alone did not generate a significant effect on any parameter or period of ethanol exposure when compared to the control/saline group. (**A**) At 3 mpf, the treatment with ethanol during the gastrula/segmentation and pharyngula stages decreased the social interaction (**P < 0.01; ****P < 0.0001) in comparison to control group (water/saline). These effects were recovered by AMPCP and EHNA in the gastrula/segmentation stage and by AMPCP in the pharyngula stage at 3 mpf. **(B)** No effect of ethanol treatment persisted until 12mpf. Data were expressed as mean ± SD and analyzed by two-way ANOVA for 3 mpf and one-way ANOVA for 12 mpf. Multiple analysis of group was performed using Tukey’s test (n = 10 animals were randomized assigned for experiments). Stimulus side = the side of aquarium near to the aquarium containing 15 conspecific fish.

Social interaction, measured by the time spent in the stimulus side of the aquarium (near to the conspecific fish tank) had significant effects from factors *ethanol treatment* [F_(2;81)_ = 22.69; *P* < 0.0001], *purinergic intervention* [F_(2;81)_ = 3.015; *P* < 0.0001], as well as, the interaction between these factors [F_(4;81)_ = 8.624; *P* < 0.0001] in animals at 3 mpf (Fig. 5A). At 3 mpf, animals exposed to ethanol at the gastrula/segmentation stage decreased their time spent on the stimulus side of the aquarium by 20% when compared to animals from control/saline group (*P* = 0.0084). Ethanol given to animals at pharyngula stage decreased the time spent on the stimulus side of the aquarium by 29% when the animals were at 3 mpf in comparison to control/saline group (*P* < 0.0001). These alterations were recovered by AMPCP and EHNA in those animals treated with 1% ethanol at the gastrula/segmentation period, while only AMPCP recovered this parameter in those animals treated with 1% ethanol during the pharyngula stage (Fig. 5A). At 12 mpf, the factor *ethanol treatment* had significant effect over time spent in the stimulus side of the aquarium [F_(2;27)_ = 4; *P* = 0.03], while multiple comparison of means did not indicate differences between control/saline group and ethanol-treated groups (Fig. 5B).

## Discussion

Here, we identified that ethanol exposure in the gastrula/segmentation and in the pharyngula developmental stages promotes long-term behavioural effects, such as increased anxiety, increased aggressiveness and decreased social interaction. These results resemble those seem in rodent studies and reinforce the zebrafish as an excellent animal model to evaluate long-term effects of early exposure to ethanol^11,38–42^. Those long-term effects of ethanol related to aggression and social interaction were recovered by adenosine metabolism manipulation.

Our results demonstrated that the exposure to 1% ethanol during the gastrula/segmentation or pharyngula stages did not affect morphology and had minor effect on locomotor activity of animals, in agreement with the literature^30,38,39,43^. While the methods of exposure to ethanol are a sensitive variable in the literature, these data suggest that none of the long-term behavioural outcomes were affected by locomotor or morphological impairment. Our results showed that, at least in behaviour alterations, the pharyngula stage (24–48 hpf) seems to be the period more affected by ethanol exposure. At the pharyngula stage, the heartbeat, vascularization and circulation in the yolk occur and some studies performed with rodents and zebrafish have demonstrated that ethanol can affect vasculogenesis and angiogenesis development, which can affect neuronal activity, a prerequisite to growth and behaviour development, which could contribute to the results seem here^28,44–46^.

Our results reinforce the long-term effect of ethanol on anxiety parameters. Ethanol exposure in the gastrula/segmentation and pharyngula stages caused an increase in the time spent in the upper zone of the tank at 3 mpf, indicating an increase of exploratory behaviour, which did not persist until 12 mpf. Recently, a similar study showed that 2-h-long exposure of zebrafish embryos to 1% ethanol at 24 hpf was also able to promote anxiogenic-like behaviour in adult zebrafish^39^. The exposure to moderate levels of ethanol appears to be more correlated to negative consequences in the social domain including decreased investigation and interaction, increased aggressive behaviour and altered responses to social stimuli^47^. In zebrafish, this behaviour could be related to the decrease in cortisol levels, since the hypothalamic–pituitary–interrenal axis is fundamental to stress and anxiety responses and involves the cortisol hormone^36^. While the involvement of the hypothalamic–pituitary–interrenal was not evaluated in this work, some studies showed that zebrafish exposed to low doses of ethanol during development had reduced the cortisol levels when evaluated at 6 months of age^48^.

The zebrafish is a social species, but the preference for being close to a conspecific can also be a result of aggressive behaviour, both of which may be influenced by ethanol^25,36^. To differentiate these behaviours, in addition to the mirror test, we observed increasing of aggressive behaviour by observation of biting, sprinting, attack behaviour and changes in colour pattern. Here, the social interaction was decreased, while aggressiveness was increased, at the young adult phase of animals exposed to ethanol during early phases of development. While the social interaction reduction did not continue until 12 mpf, the aggressive behaviour did persist until 12 mpf in the animals exposed to ethanol during pharyngula stage. The dose and time of exposures appears to be a determinant point to the long-term effects of ethanol exposure, since absent to mild and severe aggressive behaviour have been suggested in the literature^27,47^. Although in our results the social interaction reduction was not persistent until 12 mpf, studies have previously demonstrated that zebrafish embryos exposed to 1% ethanol for 2 h at 24 hpf had impaired social interaction responses at 24-month-old fish, not due to altered motor function or visual perception, but likely instead due to a central nervous system alteration affecting social behaviour itself^43^.

The literature suggests that behavioural abnormalities resulting from similar embryonic ethanol exposure may be primarily due to altered dopaminergic and serotoninergic mechanisms, while amino acid neurotransmitters, such as glutamate and GABA, seem also be somewhat related to long-term neurochemical effects of ethanol^11,49–51^. As all the major neurotransmitter systems are modulated by adenosine^16^, and adenosine appears as a target of ethanol^19,20^, some of the long-term effects of ethanol could also be related to disruption of adenosine modulation.

Studies suggest that the adenosinergic system can be involved in aggressive behaviour. Behavioural assessment of mice lacking adenosine A_1_ receptors displayed increased aggressiveness^52^. Also, inhibition of nucleoside transporters antagonizes aggressive behaviour in mice, implying that endogenous adenosine seems to play a role in influencing aggressive behaviour^53^. In this way, the reduction of aggressiveness in animals treated with ethanol by AMPCP could be a reflex of the reduction of adenosine modulatory effects. The reduction on social interaction by ethanol treatment regardless developmental stage, seems to be also affected by intervention in adenosine metabolism, since the inhibition by AMPCP reverted this effect in animals treated in both developmental stages with ethanol, and by EHNA in the animals treated with ethanol at gastrula/segmentation stage.

From all behavioural parameters evaluated, only the increase of aggressiveness was persistent to 12 mpf. Differences observed between juvenile adults and aged adults are hard to explain. However, the natural modifications of neurotransmission during lifetime could be enough to the rescue of cognitive impairment. In fact, situations when adenosine level increases, such as neonatal hypoxia, have been related to better cognition in aged rats^54^.

Our results showed that inhibition of ecto-5′-nucleotidase and adenosine deaminase were able to recover social interaction and inhibition of ecto-5′-nucleotidase recovered aggressive behaviours, both affected by early ethanol exposure. One possibility to explain these results is that the inhibition of these enzymes, especially ecto-5′nucleotidase, promotes an adjustment of adenosine modulation over several major neurotransmitters, since some of them were already identified as ethanol targets^50^. The other possibility is that the adenosine modulation could be one of the primary targets of ethanol, with long-lasting effects. In fact, previous works from our group^41^, using the same methodologic approach, demonstrated that ecto-5′-nucleotidase activity is increased in a persistent way when zebrafish is exposed to ethanol during gastrula/segmentation stage. The increasing ecto-5′-nucleotidase activity could elevate adenosine production, while we did not prove that^41^. So, in this sense, the inhibition of ecto-5′-nucleotidase by AMPCP, could reduce a persistent increased ecto-5′-nucleotidase, or the basal ecto-5′-nucleotidase, both contributing to alter the adenosine inhibitory modulation over major neurotransmitters related to social behaviour domain, such as dopamine and serotonin^42,50^. The participation of ADA is still unclear. The effects seem here could be a complex response to the systemically injections of EHNA over basal ADA activity, especially because ADA displays several isoforms with important intracellular effects^55^. Although, in vitro and ex vivo studies indicated that intra and extracellular adenosine deaminase are targets of ethanol^22,56^, a recent study did not register persistence to adulthood of impacts of early exposure to ethanol over adenosine deaminase activity and expression from zebrafish^41^.

In conclusion, the long-term effects over behaviour from early ethanol exposure can be reversed by inhibition of ecto-5′-nucleotidase and, lesser, by inhibition of adenosine deaminase in adult zebrafish. The reflex of these results over adenosine modulation should be expanded by further studies conducted to identifying the contribution of nucleoside transporters, adenosine receptors functionality and impact over major neurotransmitters modulated by adenosine in regard to these long-lasting consequences of ethanol.

## Methods

### Animals

Adult zebrafish (*D. rerio)* (Tübingen background) from the colony held at our laboratories (9–12 mpf [months post-fertilization], measuring 3.4 ± 0.5 cm; 2:1 male/female ratio) were used for obtaining fertilized eggs following the protocols of our laboratory as already published^38,41^. Briefly, animals were housed in a re-circulating system equipped with mechanical and biological filtration at a temperature of 28 °C and pH of 7.4. After mating, eggs were collected and kept in water for maintenance (water from reverse osmosis reconstituted with marine salt [Instant Ocean, Blacksburg, VA] at 0.4 parts per thousand) on a 14:10 h light/dark cycle. After the beginning of the swimming phase, larval zebrafish were transferred to aquariums and kept until 3 or 12 months of age at proper fish densities (6–9 dpf [days post-fertilization]: 16 fish/L; 18–22 dpf: 10 fish/L; 45 dpf or more: 5 fish/L). Animals were fed three times a day with commercial flakes (Tetra, NC, USA) and supplemented with live brine shrimp.

Animals were cryoanesthetized using flocked ice immersion with water with controlled physicochemical parameters at a temperature of about 2 °C, followed by euthanasia through decapitation. All procedures were in accordance with the National Institute of Health (NIH) Guide for the Care and Use of Laboratory Animals (NIH Publications No. 80–23) and were approved by the Ethics Committee for the animal use (CEUA) from the Pontifical Catholic University of Rio Grande do Sul (15/00468—CEUA PUCRS).

### Ethanol treatment

Embryos of zebrafish were exposed to 1% (v/v) ethanol (diluted in the maintenance water) in two distinct developmental stages: gastrula/segmentation [5–24 hpf (hours post-fertilization)] or pharyngula (24–48 hpf)^41^. The 1% ethanol solution (171 mM) could be considered high in comparison to human levels, which often reach 66 mM^57^. However, some investigators identified that the egg chorion acts as an important barrier, since the ethanol concentration inside the egg (6–48 hpf) is 2.7–6.2-fold lower than ethanol media levels^5,58,59^. The animals were exposed to ethanol in groups of 50 embryos, kept in Petri dishes. After the gastrula/segmentation or pharyngula stages, animals were maintained in drug-free water until they reach 3 or 12 mpf, two ages when the animals are mature and present preserved locomotor activity^59^.

### Exposure to enzymatic inhibitors AMPCP or EHNA

To investigate the effect of adenosine metabolism over behavioural impacts of early ethanol exposure, we used the specific inhibitors of adenosine deaminase, EHNA (erythro-9-(2-hydroxy-3-nonyl)adenine hydrochloride), which could avoid adenosine degradation, and AMPCP (α,β-Methyleneadenosine 5′-diphosphate) an inhibitor of 5′-nucleotidase, which could inhibit adenosine formation by AMP catabolism. The pharmacological treatments (AMPCP at 150 mg/kg or EHNA at 100 mg/kg) of adults were performed by intraperitoneal (ip) injection with the use of a Hamilton microliter syringe in a volume of 10 μL per animal (20 mL/kg), following literature using zebrafish^60^. Prior to the injection, the adult animals were anesthetized in tricaine solution (MS-222; 100 mg/L)^60,61^. Behavioural analysis was performed 30 min after drug exposure. Control animals received saline.

### Locomotion and anxiety assessment

Animals at 3 or 12 mpf had their locomotor and anxiety parameters performed after 30 min of AMPCP, EHNA or saline injections. Zebrafish were individually placed in a tank (30 × 15 × 10 cm, length × height × width). After 30 s of adaptation, the zebrafish activity was recorded for 5 min and analysed using the video tracking system Ethovision XT8 (Noldus, Netherlands). The locomotor parameters analysed were distance travelled (m) and mean speed (m/s; assessed only during moving time). Exploratory behaviour, an indicator of anxiety, was assessed as the time spent in the top section of tank and was virtually divided into two horizontal lines (bottom and upper zones)^36,59,60^ (Supplementary Fig. S1A). The locomotion and anxiety were evaluated individually for each drug (AMPCP or EHNA) in each stage (gastrula/segmentation or pharyngula) and for each control/saline (no ethanol added) with 10 animals per group. Randomized and independent groups of animals were used for locomotor and anxiety test.

### Aggressiveness assessment

At 3 or 12 mpf, aggressive behaviour was analysed in control and ethanol-treated animal 30 min after injection of AMPCP, EHNA or saline (n = 10). Individually, the fish were placed in a single tank (30 × 15 × 10 cm, length × height × width) with a mirror (22 cm) positioned in the back of the tank forming a 22.5° angle^25,60,62^ (Supplementary Fig. S1B). The fish were habituated for 30 s, after which the locomotion was video recorded for 8 min^59^. The tank was virtually divided into four equal sections, from which the time spent in each zone was analysed, with the time spent in the zone near the mirror being the sign of aggressive behaviour. The activity was analysed using the video tracking system Ethovision XT8 (Noldus, Netherlands). Additionally, observations of aggressive behaviour, like biting, sprinting and changes in colour pattern, were also made. Usually, attack behaviour is a characteristic short bout of fast swimming directed towards the opponent and is sometimes accompanied by opening the mouth and biting^62^. Randomized and independent groups of 10 animals were used for aggressiveness test.

### Social interaction assessment

At 3 or 12 mpf, the social interaction behaviour of control and ethanol-treated animals was analysed 30 min after AMPCP, EHNA or saline injections (n = 10). Three tanks (30 × 15 × 10 cm, length × height × width) were placed side by side: the far-left tank was left empty, the one in the middle held the test animals and the far right held 15 stimuli conspecific fish^36^ (Supplementary Fig. S1C). Zebrafish were placed individually in the test aquarium, and after 5 min habituation, their social interaction was recorded for 10 min. The tank with the test fish was separated into two equal sections, with the social interaction indicator defined as the time spent on the side closest to the stimulus fish. All data were analysed using the video tracking system Ethovision XT8 (Noldus, Netherlands). Randomized and independent groups of 10 animals were used for social interaction test.

### Statistical analysis

The normality of data was checked using the D’Agostino & Pearson Omnibus normality test. Two-Way Analysis of Variance (ANOVA) was used to compare groups considering as factors the ethanol exposure (no ethanol *versus* ethanol on gastrula stage *versus* ethanol on pharyngula stage = factor *ethanol treatment*) and the purinergic drug exposure (no drug *versus* AMPCP *versus* EHNA = factor *purinergic intervention*) at adult life. The effect of water *versus* ethanol treatment at 12 mpf animals for time on upper zone (anxiety measure) and time in the stimulus side (social interaction measure) was analysed by One-Way ANOVA, since the AMPCP and EHNA were not tested for this analysis at this age. The multiple analyses of the means were analysed by Tukey’s multiple comparisons test in behaviour and locomotors parameters when appropriated. The significance levels were attributed at *P* < 0.05. Data were expressed as mean ± standard deviation (SD).

## Supplementary information

Supplementary Figure.

Supplementary Table.
