# Supplementary material for: Inhibition of ecto-5′-nucleotidase and adenosine deaminase is able to reverse long-term behavioural effects of early ethanol exposure in zebrafish (Danio rerio)
Source: Sci Rep. 2020 Oct 20;10:17809. doi: 10.1038/s41598-020-74832-0 (PMC7576130; doi:10.1038/s41598-020-74832-0)
Supplement: Supplementary file 1 — Supplementary Figure. [file 41598_2020_74832_MOESM1_ESM.pdf]

Title: **Inhibition of ecto-5'-nucleotidase and adenosine deaminase is able to reverse long-term behavioural effects of early ethanol exposure in zebrafish (Danio rerio**

Authors: Aline Haab Lutte, Julia Huppes Majolo, Rosane Souza Da Silva

Supplementary Figure S1A

Anxiety measurement apparatus

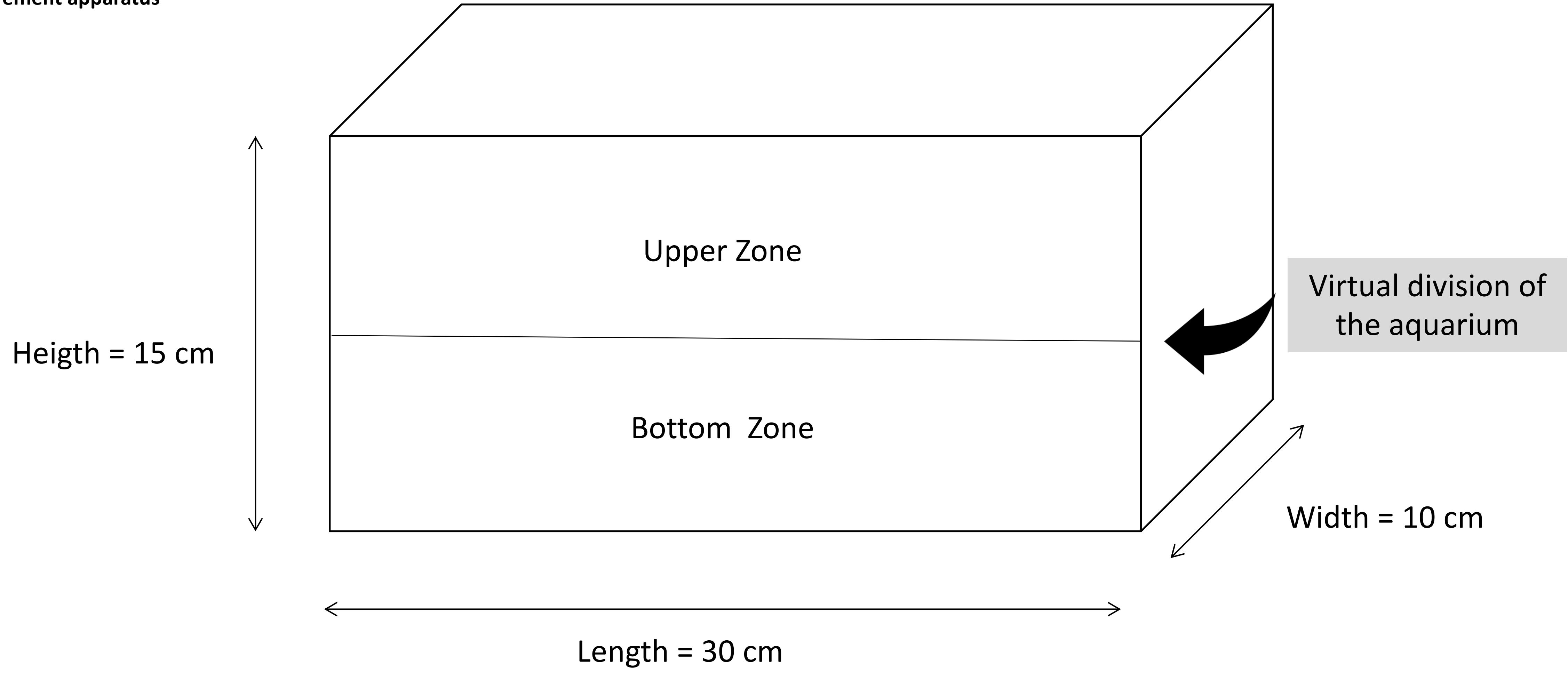

Supplementary figure S1A: Zebrafish were individually placed in a tank (30 × 15 × 10 cm, length × height × width). After 30 sec of adaptation, the zebrafish activity was videorecorded for 5 min. Exploratory behaviour, an indicator of anxiety, was assessed as the time spent in the top section of tank and was virtually divided into two horizontal lines (bottom and upper zones).

Title: **Inhibition of ecto-5'-nucleotidase and adenosine deaminase is able to reverse long-term behavioural effects of early ethanol exposure in zebrafish (Danio rerio**

Authors: Aline Haab Lutte, Julia Huppes Majolo, Rosane Souza Da Silva

Supplementary Figure S1B

Aggressive behaviour measurement apparatus

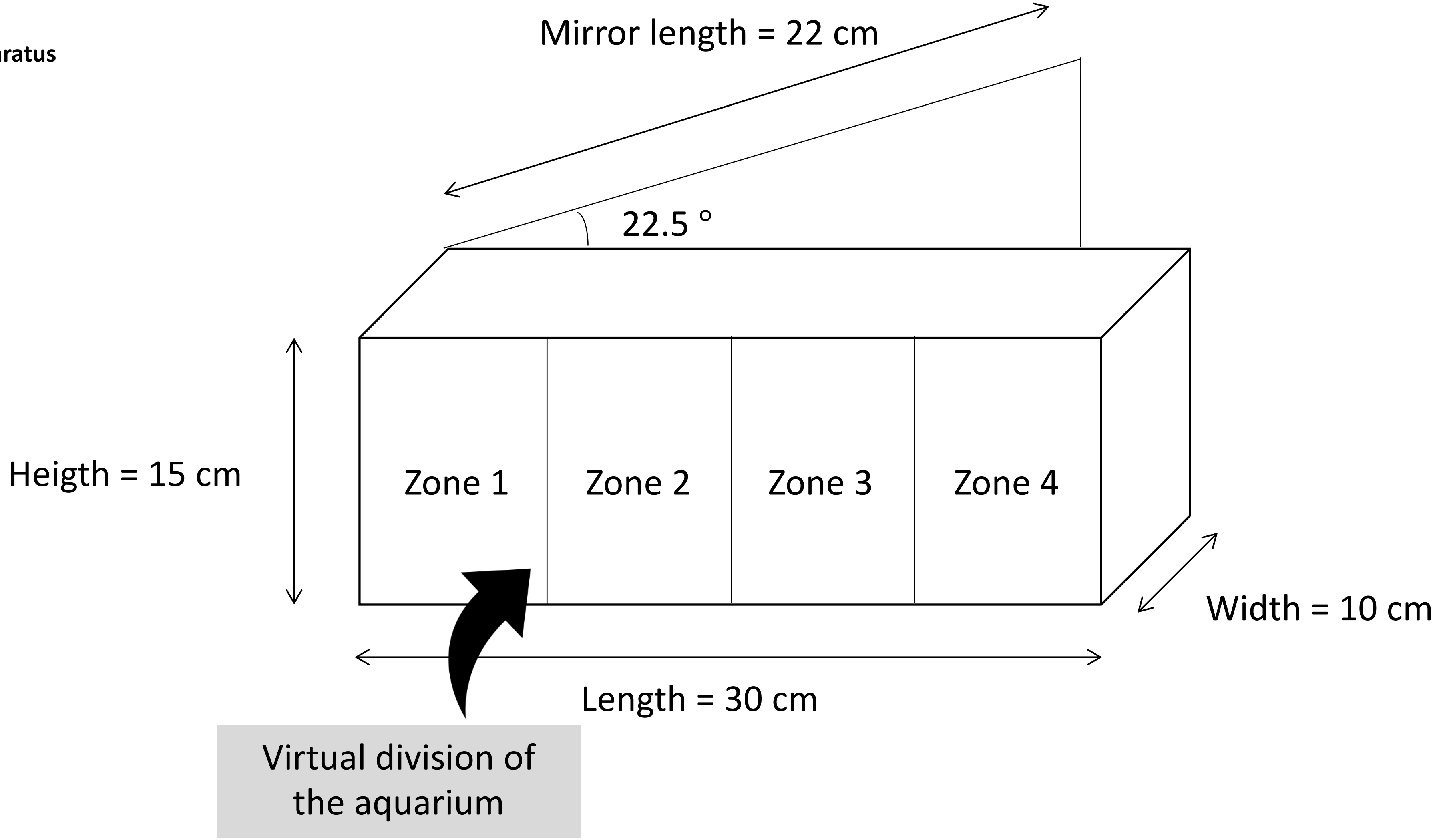

Supplementary figure S1B: Zebrafish were individually placed in a tank (30 × 15 × 10 cm, length × height × width) with a mirror (22 cm) positioned in the back of the tank forming a 22.5° angle. The fish were habituated for 30 sec, after which the locomotion was video recorded for 8 min. The tank was virtually divided into four equal sections, from which the time spent in each zone was analysed, with the time spent in the zone near the mirror being the sign of aggressive behaviour.

Title: **Inhibition of ecto-5’-nucleotidase and adenosine deaminase is able to reverse long-term behavioural effects of early ethanol exposure in zebrafish (Danio rerio**

Authors: Aline Haab Lutte, Julia Huppes Majolo, Rosane Souza Da Silva

Supplementary Figure S1C

Social interaction behaviour measurement apparatus

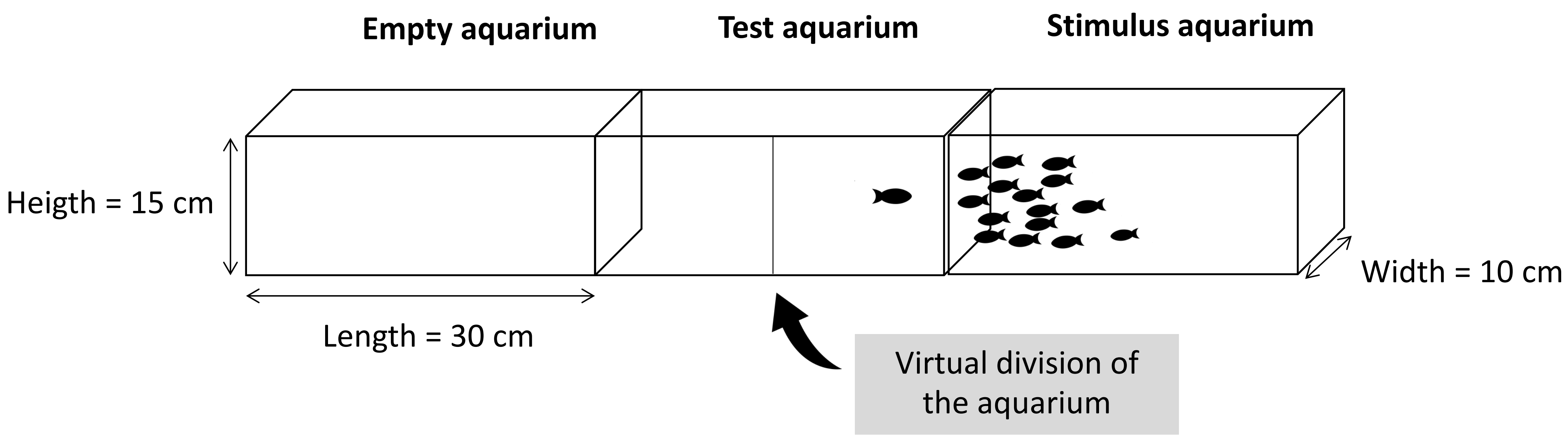

Supplementary figure S1C: Three tanks (30 × 15 × 10 cm, length × height × width) were placed side by side: the far left tank was left empty, the one in the middle held the test animals and the far right held 15 stimulus conspecific fish. Zebrafish were placed individually in the test aquarium, and after 5 min of habituation, their social interaction was videorecorded for 10 min. The tank with the test fish was separated into two equal sections, with the social interaction indicator defined as the time spent on the side closest to the stimulus fish.
