# Supplementary material for: Inhibition of ecto-5′-nucleotidase and adenosine deaminase is able to reverse long-term behavioural effects of early ethanol exposure in zebrafish (Danio rerio)
Source: Sci Rep. 2020 Oct 20;10:17809. doi: 10.1038/s41598-020-74832-0 (PMC7576130; doi:10.1038/s41598-020-74832-0)
Supplement: Supplementary file 2 — Supplementary Table. [file 41598_2020_74832_MOESM2_ESM.pdf]

Title: Modulation of adenosine metabolism is able to reverse long-term behavioural effects of early ethanol exposure in zebrafish (*Danio rerio*)

Authors: Aline Haab Lutte, Júlia Huppes Majolo, Rosane Souza Da Silva

Supplementary table 1: Aggressive behaviors

	**3 mpf**	**Treatment during early development**								
		**Ethanol** Gastrula			**Ethanol** Pharyngula			**Water**		
	**Adult treatment**	Saline	EHNA	AMPCP	Saline	EHNA	AMPCP	Saline	EHNA	AMPCP
**Aggressive behavior parameters**	Biting	0.3±0.3	0	1	$6.3\pm0.8^{***}$	$7\pm0.6^{***}$	1.33±0.9	0	0	0
	Sprinting	0	0	0	3.33±0.9	$5\pm0.6^{***}$	0	0	0	0
	Changes in color patterns	0	0	0	0.66±0.3	1±0.6	0	0	0	0

	**12 mpf**	**Treatment during early development**					
		**Ethanol** Gastrula		**Ethanol** Pharyngula		**Water**	
	**Adult treatment**	Saline	AMPCP	Saline	AMPCP	Saline	AMPCP
**Aggressive behavior parameters**	Biting	0	0	$5.6\pm1.2^{***}$	1.0±0.6	0	0
	Sprinting	0	0	$4.0\pm1.15^{**}$	0.66±0.33	0	0
	Changes in color patterns	0	0	$2.33\pm0.3^{***}$	0	0	0

Table Legend

Table 1: Mean ±S.E.M of times that fish displayed biting, sprinting or changes in colour pattern during 8 minutes of evaluation. This analysis was performed at 3 and 12 months post-fertilization animals. Randomized and independent groups of 10 animals were used for behavioural test. **$P<0.01$ and ***$P<0.001$ in comparison to control group (water/saline).
